# Salient distractors open the door of perception: alpha desynchronization marks sensory gating in a working memory task

**DOI:** 10.1038/s41598-020-76190-3

**Published:** 2020-11-05

**Authors:** Zsuzsanna Fodor, Csilla Marosi, László Tombor, Gábor Csukly

**Affiliations:** Department of Psychiatry and Psychotherapy, Semmelweis University, Balassa utca 6, Budapest, 1083 Hungary

**Keywords:** Cognitive control, Working memory, Biomarkers, Human behaviour

## Abstract

Focusing attention on relevant information while ignoring distracting stimuli is essential to the efficacy of working memory. Alpha- and theta-band oscillations have been linked to the inhibition of anticipated and attentionally avoidable distractors. However, the neurophysiological background of the rejection of task-irrelevant stimuli appearing in the focus of attention is not fully understood. We aimed to examine whether theta and alpha-band oscillations serve as an indicator of successful distractor rejection. Twenty-four students were enrolled in the study. 64-channel EEG was recorded during a modified Sternberg working memory task where weak and strong (salient) distractors were presented during the retention period. Event-related spectral perturbation in the alpha frequency band was significantly modulated by the saliency of the distracting stimuli, while theta oscillation was modulated by the need for cognitive control. Moreover, stronger alpha desynchronization to strong relative to weak distracting stimuli significantly increased the probability of mistakenly identifying the presented distractor as a member of the memory sequence. Therefore, our results suggest that alpha activity reflects the vulnerability of attention to distracting salient stimuli.

## Introduction

Visual working memory enables us to temporarily hold and use the information for further processing but is limited in capacity. A crucial factor in determining working memory capacity is the effective filtering of irrelevant and distracting stimuli^1,2^, even over the enhancement of task-relevant information^3^. Former studies suggested that alpha-band oscillations play an important role in distractor filtering^4–6^ and theta-band oscillations have been linked to successful memory manipulation and general cognitive control, which are also essential components of efficient filtering^7,8^. However, the neural mechanisms and the oscillatory hallmarks of distractor inhibition are not fully understood^9^.

Oscillatory activity in the alpha frequency band has been linked to top-down modulation of attention^10,11^ and distractor filtering by several studies^4–6^. Specifically, it has been suggested that alpha oscillation plays an important role in the top-down controlled gating of task-relevant information by the inhibition of task-irrelevant neural populations^12,13^ by rhythmic synchronization through coherence^14^. Studies supporting this notion report that the scalp distribution of alpha-band power follows the focus of spatial attention, by decreasing over task-relevant areas and increasing over brain regions that represent distracting information^6,15–19^, and confirmed a causal relationship between alpha-band oscillations and the attentional suppression of sensory information by using transcranial magnetic stimulation^20^. However, it has been pointed out recently, that many of the existing evidence that links alpha activity with spatial attention is compatible with an account in which alpha activity supports target selection via signal enhancement^21^.

Numerous former EEG studies confirmed that increasing cognitive demand enhances frontal theta oscillatory activity^22–26^. However, recent studies pointed out that theta oscillations over the frontal cortex might reflect the realization of the need for cognitive control in goal-directed high-level cognitive processes such as working memory retention^7,27^.

Recent studies pointed out that to clarify the role of oscillatory activity in distractor filtering it should be taken into account whether participants can shift their attentional focus before the appearance of the distractors or not, as this influences the filtering process, recruit distinct mechanisms and involve different underlying neural structures^28–30^. Most of the tasks used in previous studies used attentional guidance by presenting targets and distractors bilaterally around a central fixation point along with a cue indicating the to-be memorized hemifield while presenting distractors on the other side of the screen^6,30–32^.

In contrast, there is far less and mixed evidence about the role of theta and alpha-band oscillatory modulation in the rejection of task-irrelevant stimuli when distractors cannot be attentionally avoided^29,30,32^. Moreover, in most of the previous studies, targets and distractors were displayed simultaneously, and only a few of them applied a sequential presentation^33^ or used a design, that allows the dissociation of the oscillatory activity related to the processing of targets and distractors^34^. Furthermore, there are very few studies that took into account that the saliency of the distracting stimuli might potentially influence the oscillatory dynamics^32^.

We aimed to examine directly whether theta and alpha-band oscillations serve as an indicator of successful distractor rejection. We applied the Sternberg working memory task with independent manipulation of memory set size and distractor saliency, which allows the temporal separation of the encoding and the retention condition including the presentation of distractors. Weak and strong (salient) distractors following memory sequences were presented at the same location as the targets to keep them in the focus of attention.

As previous studies suggest, that cognitive control-related modulations affect the induced oscillations^7,35^ we analyzed the event-related spectral perturbation (ERSP), which can capture the non-phase locked as well as the evoked oscillations^36^.

Based on previous research we assumed that the salient distracting stimuli will induce enhanced alpha^29,32^ and theta oscillations^32^ compared to the weak (non-salient) distractors, while the increased need for cognitive control related to the rejection of distractors and especially related to the inhibition of salient distractors will enhance theta oscillatory activity, especially in the frontal region^7,27,35^.

## Materials and methods

### Participants

The study was carried out in the Department of Psychiatry and Psychotherapy, Semmelweis University, Budapest, Hungary. Altogether 24 students of Semmelweis University (10 females, mean age 24 ± 2.15 years) were enrolled in the study. All participants were right-handed except for one ambidextrous subject. Selection criteria were no history of head injury, stroke, mental retardation, epileptic seizure, acute psychiatric disorder, or substance dependence. All procedures contributing to this work comply with the ethical standards of the relevant national and institutional committees on human experimentation and with the Declaration of Helsinki. The National Scientific and Ethical Committee, Budapest, Hungary approved the research. Participants gave their written informed consent before the procedures.

### EEG stimuli and procedures

During the EEG recording, participants were seated in a dimly lit, sound-attenuated room. All participants had normal or corrected-to-normal vision. Subjects performed a modified version of the Sternberg task^37^, an extensively used test of working memory^38,39^. The original task was adjusted to assess the effect of distractors on working memory retention. The stimuli consisted of white, green, and blue letters, Arial font, point size 60, that were presented on a computer screen at approximately 50 cm with Presentation 13.0 software (Neurobehavioral Systems, Inc.; Albany, CA).

**Figure 1:**
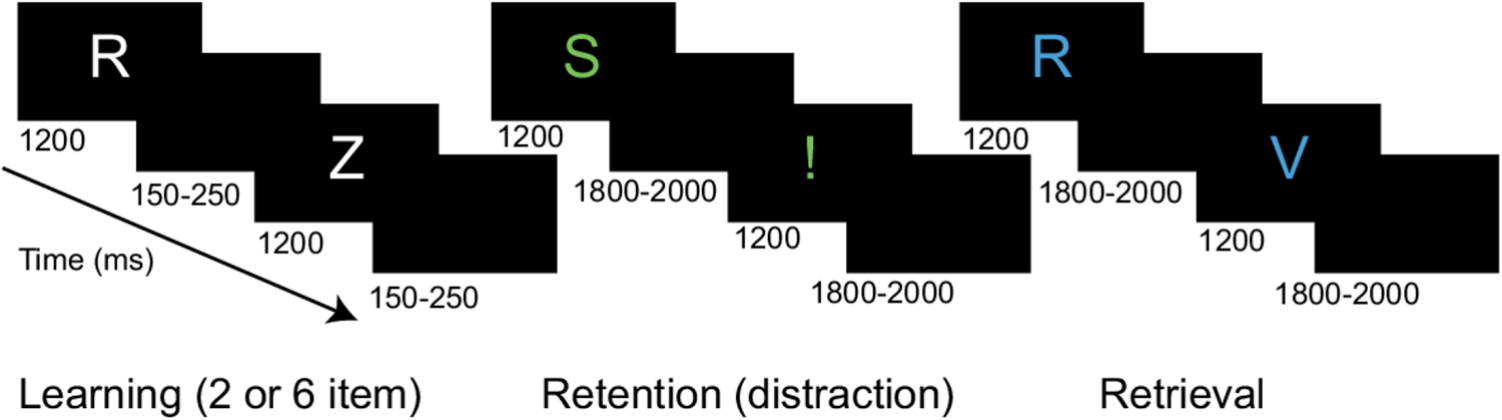
The modified Sternberg working memory task. Following the memory sequence, during the retention period of the task, a strong (salient, green consonant) and a weak (non-salient, green exclamation mark) distractor was presented in a randomized order. The Figure was prepared by using Adobe Illustrator CC 2018 software.

At the onset of each trial, two or six randomly selected white consonants appeared sequentially in the middle of the screen for 1200 ms, separated by a blank screen for 150–250 ms. During the retention period, two green-colored distractors were presented in a randomized order for 1200 ms separated by a blank screen for 1800–2000 ms: a green exclamation mark (weak distractor) and a green consonant that was not part of the previous learning sequence (strong distractor). In the retrieval condition, two blue consonants were presented for 1200 ms separated by a blank screen presented for 1800–2000 ms (Fig. 1). Subjects were instructed to indicate by clicking the mouse buttons (yes-right / no-left) whether the probe letter was part of the presented sequence. The length of the learning sequence, the order of the distractors, and the response assignment were counterbalanced across 144 trials. Efficiency was measured by response accuracy. Working memory capacity was estimated using Cowan's K formula^40^ for each set-size: K = set size × (hit rate – false alarm rate). Participants completed the task in six equal parts separated by a 3-min rest period. It was carefully monitored that the participants understood the instructions and stayed alert during the session.

### EEG recording and processing

EEG was recorded from DC with a low-pass filter at 100 Hz using a high-density 64-channel BioSemi ActiveTwo amplifier^41^. Electrode caps had an equidistant-layout and covered the whole head. Eye movements were monitored with EOG electrodes placed below the left and above the right external canthi. Data were digitized at a sampling rate of 1024 Hz. Built-in and self-developed functions as well as the freeware EEGLAB toolbox^42^ in the MATLAB (MathWorks, Natick, MA) development environment were used for subsequent off-line data analyses. EEG was re-referenced to the common average potential and filtered off-line between 0.5 and 45 Hz using zero-phase shift forward, and reverse IIR Butterworth filter.

Epochs from 500 ms pre-stimulus to 1350 ms post-stimulus were extracted from the continuous EEG and corrected for the pre-stimulus baseline adjusted to the presentation of the last items of the learning sequences and of the weak and strong distractors during the retention period.

Removal of muscle, blinking, and eye movement artifacts (detected by EOG) were performed by ADJUST^43^, and ICA (Independent Component Analysis) based automatic artifact detector. Furthermore, epochs with a voltage exceeding ± 100 μV on any channel were rejected from the analysis.

After artifact rejection, the average number of trials was 132.2 (SD = 11.2) for the learning, 132.3 (SD = 11.0) for the weak distractor, and 132.1 (SD = 13.3) for the strong distractor condition, respectively. For the detailed analysis, we selected 4 scalp regions (frontal, central, right parieto-occipital, left parieto-occipital).

### EEG data analysis

Stimulus-related theta (4.1–7 Hz) and alpha (7.1–13 Hz) activity changes were measured by the ERSP, providing a 2-D representation of the mean change in spectral power (in dB) from baseline^36^. Beyond conventional event-related analytical approaches, ERSP can capture event-related as well as non-phase locked (induced) oscillatory activity. Since distractor filtering is a relatively time-consuming complex cognitive process involving the cooperation of distant cortical regions^28^ and especially, because it has been shown that cognitive control-related processes modulate the induced oscillatory activity^7,35^ we applied the ERSP approach in our analysis. To compute the ERSP, baseline spectra are calculated from the EEG immediately preceding each event. The epoch is divided into brief, overlapping data windows, and a moving average of the amplitude spectra is created. Each of these spectral transforms of individual response epochs is then normalized by dividing by their respective mean baseline spectra. Normalized response transforms for many trials are then averaged to produce the average ERSP, plotted as relative spectral log amplitude on a time-by-frequency plane^36^ (see details in the Supplementary Information).

The analysis was performed on epochs extending from 500 ms before to 1350 ms after stimulus onset in learning, weak distractor, and strong distractor condition, respectively in the 1.5–30 Hz frequency range. The sliding window was 400 ms wide, and it was applied 200 times with an average step size of 6.8 ms. No zero padding was applied. The analyzed time interval lasted from 282 ms before to 1133 ms after stimulus onset. Dynamical changes in oscillatory activity were studied by computing ERSPs for each trial, then averaging them separately for learning, weak distractor, and strong distractor condition^44,45^.

The ERSP time–frequency matrices were baseline corrected by the average power calculated from the 500 to 200 ms pre-stimulus period for the assessment of the modulatory effect of distractor saliency and memory load to oscillatory activity during the retention period. To compare ERSP across conditions a second analysis was conducted, using the average power calculated from the 500 to 200 ms period before the first item of the learning sequences as a common baseline for the retention and learning conditions. In this analysis, epochs of only the last items of the learning sequences were included to minimize memory load-related differences between the conditions. Note that in this case, baseline values did not originate from the pre-stimulus period and for this reason, the sum of the baseline data points is not necessarily zero. However, thus we could exclude the possibility of finding differences in ERSP caused by differences in baseline activity in the learning and retention period.

Former studies found memory load-related and distractor saliency-related modulation of the alpha activity from 500 to 1300 ms following the stimulus presentation^29,30,46^. Therefore, based on these prior results and adjusted to the end of the desynchronization in the weak distractor condition we selected the 700–1000 ms time window in the alpha band for further analysis to select a time window past the initial encoding of the stimulus. For the assessment of the theta ERSP, we selected the 300–600 ms time window, as previous studies detected cognitive control-related midfrontal theta power modulation in this time window^35,47^.

Mean ERSP values were calculated by averaging across electrodes within scalp regions to further attenuate noise. As we aimed to identify and highlight robust distractor rejection-related modulation of electrophysiological activity, every scalp region was involved in the regional analysis. Parieto-occipital region was divided to right and left side to avoid the conflation of the results due to averaging across too many electrodes and since the task-related stimuli were letters, which are generally processed in brain areas located in the left hemisphere^48–50^, which may cause the lateralization of the task-related modulation^51,52^. To avoid the loss of information due to the aggregation of multiple electrodes, and based on prior studies of cognitive control that selectively examined frontal-midline electrodes^27,35,53^ we also analyzed the theta activity of the Fz electrode separately.

### Statistical analysis

Behavioral results between the 2- and 6-item conditions were compared with paired t-test. The different effects on ERSP in the distractor conditions were tested by three-way analyses of variance (ANOVA) of the distractor type (strong vs. weak) × memory load (2-item vs. 6-item) × region while for the comparison of the learning and distractor conditions two-way ANOVA of the condition (learning vs. strong distractor vs. weak distractor) × region was used. Normal distribution of variables was tested using the Kolmogorov–Smirnov test. Post-hoc pairwise contrasts were conducted to investigate the interactions. Since post-hoc comparisons were evaluated over four regions, Hochberg correction for multiple comparisons was applied to the post-hoc contrasts^54,55^. To characterize the magnitude of the reported effects we reported the values of effect size^56^.

The associations between the behavioral results and the task-related modulation of ERSP in the frontal region were assessed by Pearson correlation. Alpha reactivity was characterized by the difference of alpha ERSP in the weak and strong distractor condition (alpha ERSP_weak distractor_—alpha ERSP_strong distractor_) in the 700–1000 ms time window, while theta reactivity was marked by the difference of theta ERSP in the learning and strong distractor condition in the 300–600 ms time window (theta ERSP_learning_—theta ERSP_strong distractor_).

In the correlational analysis, we focused on the frontal region. Former fMRI studies showed that distraction suppression activates the prefrontal cortex specifically^57–60^. Moreover, former EEG studies identified the prefrontal cortex as the main source of top-down attentional control of visual cortical activity during working memory retention mediated by alpha oscillations^61^ and the origin of theta activity which is modulated by the need for cognitive control^7,27^, therefore we hypothesized that the task-related modulations of frontal alpha- and theta-band oscillations will reflect the regulation of attentional and cognitive control.

Figures were prepared by using MATLAB and Statistics Toolbox Release 2017a and SAS 9.4 software.

## Results

### Behavioral results

Response accuracy of the participants was significantly decreased under high memory load compared to low memory load (mean_2-items_ = 96.25% SD = 3.25, mean_6-items_ = 92.02% SD = 5.06, t_1,23_ = 5.9, *p* < 0.0001, Cohens’s D = 1.0). Furthermore, subjects made significantly more “distractor commission error” (i.e. participant mistakenly identifies the previously presented distractor as a member of the memory sequence) under high memory load, when they mistakenly identified the strong distractor as part of the learning sequence (mean_2-item, distractor commission error_ = 1.27% SD = 1.14, mean_6-item, distractor commission error_ = 2.50% SD = 2.09, t_1,23_ = − 2.84, *p* = 0.009, Cohens’s D = 0.8).

### Alpha ERSP in weak and strong distractor conditions

**Figure 2:**
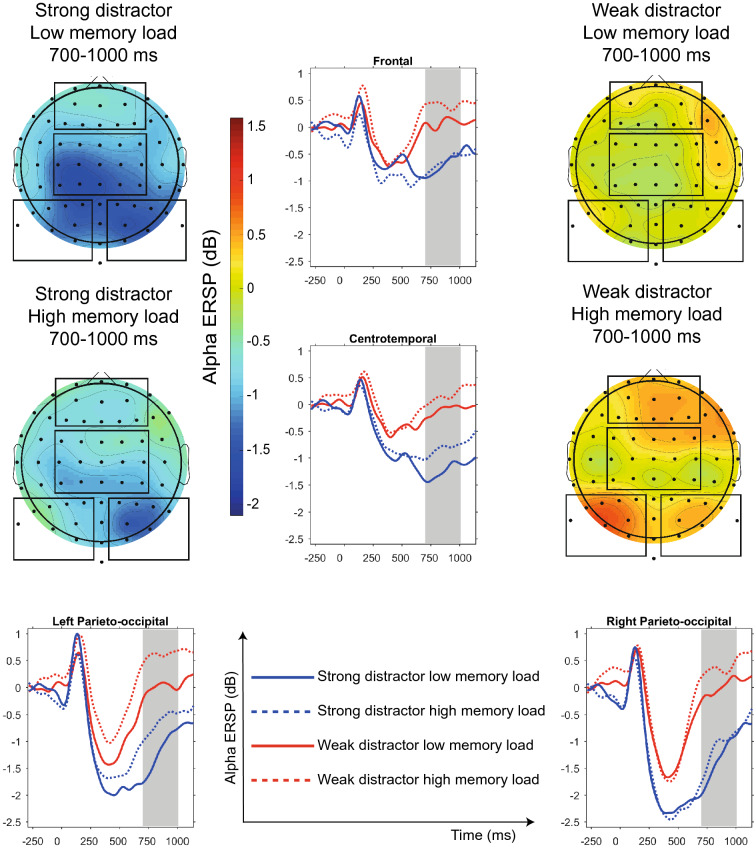
Alpha ERSP in strong and weak distractor conditions. Alpha event-related spectral perturbation (ERSP) in strong and weak distractor conditions (700–1000 ms time window highlighted) and scalp topography of the alpha ERSP groups in the 700–1000 ms time window. The Figures was prepared by using MATLAB and Statistics Toolbox Release 2017a.

Alpha desynchronization (ERD: decrease in alpha ERSP) was observable (Fig. 2) during the presentation of weak as well as of strong distractors, however, a significantly stronger alpha ERD (F(1,23) = 27.4, *p* < 0.0001, Cohen’s D = 1.0) was detected in the strong distractor condition compared to the weak distractor condition. Furthermore, alpha ERD was significantly weaker following sequences with high memory load in contrast to low memory load (F(1,23) = 10.8, *p* = 0.0032, Cohen’s D = 0.3). Moreover, region had a significant effect on alpha ERD (F(3,23) = 11.4, *p* < 0.0001) with maximum alpha ERD in the right parieto-occipital region during the strong distractor conditions following a low memory load set. Interaction of distractor type and region (F(3,23) = 4.4, *p* = 0.01) and memory load and region (F(3,23) = 5.1, *p* = 0.007) had a significant effect as well. Post hoc analyses of these interactions revealed significant memory load-related modulation of alpha ERSP (ERD_low memory set_ > ERD_high memory set_) in the central (t = 4.1, df = 23, *p* = 0.0004, Cohen’s D = 0.3) and left parieto-occipital (t = 4.3, df = 23, *p* = 0.0003, Cohen’s D = 0.4) region, and significant distractor type-related modulation of alpha ERSP (ERD_strong distractor_ > ERD_weak distractor_) in every region (t > 3.8, *p* < 0.05, Cohen’s D >  = 0.9), with a maximum difference between distractor types at the central region (t = 7.0, *p* < 0.0001, Cohen’s D = 1.2) These differences remained significant even after correction for multiple comparisons.

### Alpha ERSP in learning and distractor conditions

Condition had a significant effect on alpha ERSP (F(2,23) = 19.4, *p* < 0.0001). Significantly decreased alpha ERSP was found compared to the weak distractor condition in the learning (t = 5.7, df = 23, *p* < 0.0001, Cohen’s D = 0.4) and in the strong distractor condition (t = 5.3, df = 23, *p* < 0.0001, Cohen’s D = 0.4), while there was no significant difference in alpha ERSP between the learning and strong distractor conditions (*p* > 0.05, Cohen’s D = 0.01). Region (F(3,23) = 107.8, *p* < 0.0001) and interaction of condition and region (F(6,23) = 14.2, *p* = 0.0001) had a significant effect as well. Post hoc analyses of this interactions revealed significant increase of alpha ERSP during the weak distractor condition relative to the learning and strong distractor condition in every region (t > 3.43, *p* ≤ 0.0002) with a maximum difference between learning and the weak distractor condition at the right parieto-occipital region (t = 7.23, p < 0.0001, Cohen’s D = 0.6), which remained significant after correction for multiple comparisons, while alpha ERSP in the learning and strong distractor condition did not show difference in any region (*p* > 0.05) (Supplementary Fig. 2).

### Correlational analysis of distractor related alpha oscillatory activity

To explore the relation between attentional control-related modulation of frontal alpha oscillatory activity and task performance, we characterized distractor saliency-related alpha reactivity by the difference of ERSP in the strong and weak distractor condition. Alpha reactivity in the frontal region correlated significantly with mean working memory capacity (k_mean_, r = 0.44, *p* = 0.03). Moreover, significant negative correlations were found between the number of “distractor commission errors” and alpha reactivity in the frontal region (r = − 0.47, *p* = 0.02) (Fig. 4) However, these results did not remain significant after correction for multiple comparisons.

### Theta ERSP in weak and strong distractor conditions

Theta synchronization (ERS: increase in theta ERSP) was observable during the presentation of weak as well as of strong distractors, however, theta ERS did not differ significantly in the strong and weak distractor conditions in the 300–600 ms time window (*p* > 0.05, Cohen’s D = 0.14). Moreover, memory load did not have a significant modulatory effect on theta ERS (*p* > 0.5, Cohen’s D = 0.10) (Supplementary Fig. 1).

### Theta ERSP in learning and distractor conditions

**Figure 3:**
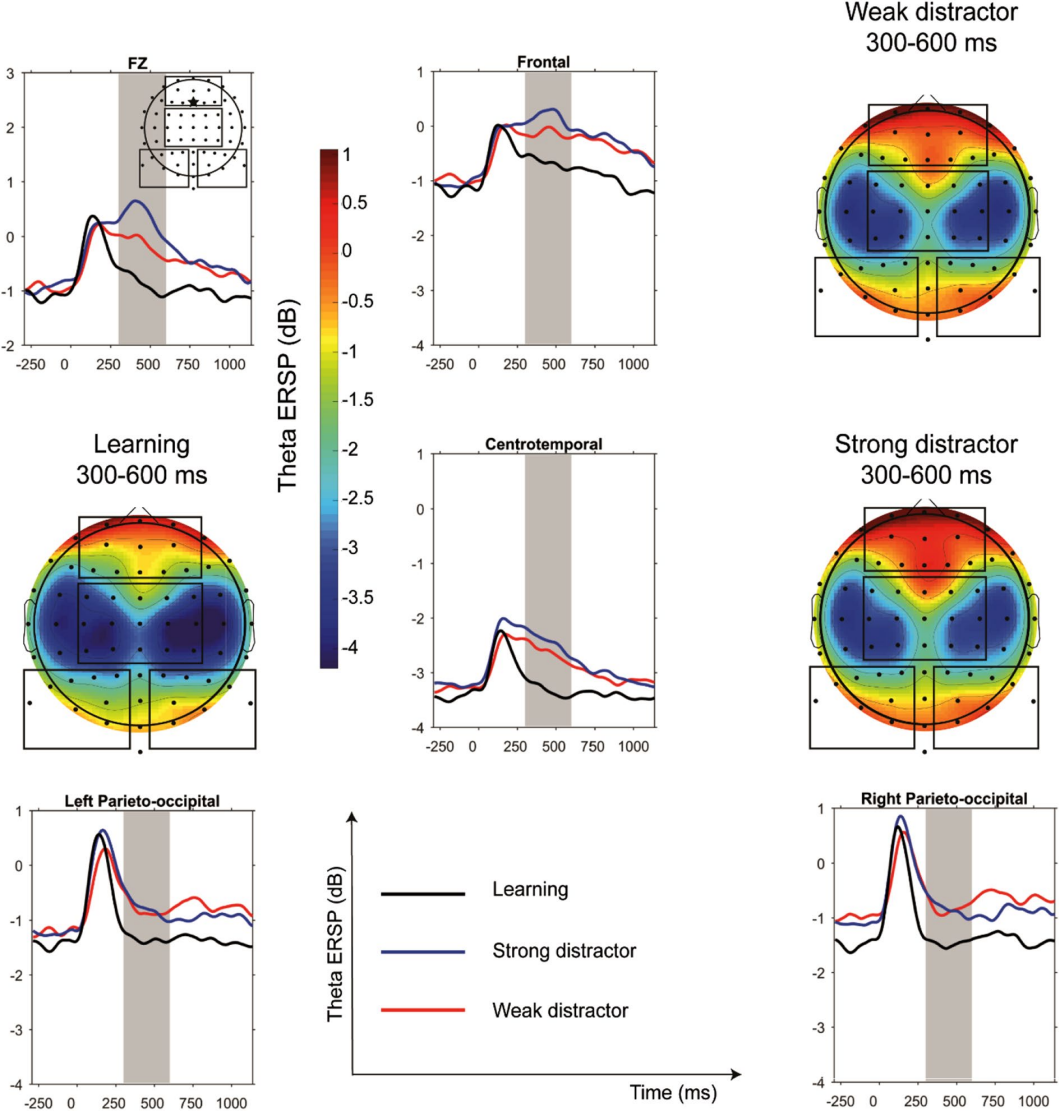
Theta ERSP in learning, strong and weak distractor conditions. Theta event-related spectral perturbation (ERSP) in learning, strong and weak distractor conditions (300–600 ms time window highlighted), and scalp topography of the theta ERSP groups in the 300–600 ms time window. The Figure was prepared by using MATLAB and Statistics Toolbox Release 2017a.

Condition (learning vs weak distractor vs strong distractor) had a significant effect on theta (4–7 Hz) ERSP (F(2,23) = 30.0, *p* < 0.0001) (Fig. 3). Significantly increased theta ERSP was found during the weak (t = 5.7, df = 23, *p* < 0.0001, Cohen’s D = 0.3) and the strong distractor condition (t = 6.7, df = 23, *p* < 0.0001, Cohen’s D = 0.3) compared to the learning condition, while there was no significant difference in theta ERSP between the weak and strong distractor conditions (*p* > 0.05 Cohen’s D = 0.06). Region (F(3,23) = 43.9, *p* < 0.0001) and interaction of condition and region (F(6,23) = 7.9, *p* = 0.0001) had a significant effect as well. Post hoc analyses of this interactions revealed significant increase of theta ERSP during the distractor conditions relative the learning condition, regardless of the distractor type in every region (t ≥ 3.78, *p* ≤ 0.0001) with a maximum difference between learning and the strong distractor condition at the central region (t = 8.45, p < 0.0001, Cohen’s D = 0.4), which remained significant after correction for multiple comparisons, while theta ERSP in the weak and strong distractor condition did not show any difference in any region (*p* > 0.05).

We analyzed theta ERSP in the same time window selectively in the Fz electrode as well. Condition had a significant effect on frontal midline theta ERSP (F(2,23) = 27.9, *p* < 0.0001) (Fig. 3). Significantly higher theta ERSP was found in the strong distractor condition compared to the weak distractor (t = 3.2, df = 23, *p* < 0.0038, Cohen’s D = 0.2) and to the learning (t = 7.1, df = 23, *p* < 0.0001, Cohen’s D = 0.5) condition. Furthermore, in the weak distractor condition a significantly increased theta ERSP was detected compared to the learning condition (t = 5.3, df = 23, *p* < 0.0001, Cohen’s D = 0.3).

### Effect of bottom-up differences of visual stimulation on the N1 ERP component and correlational analysis with the ERSP

To rule out that the detected distractor rejection-related modulatory effects were driven by differences in low-level features, we examined the N1 component which is sensitive to physical properties of the stimuli based on former studies^62–65^ and its correlation with alpha and theta ERSP. The physical differences between the letters and the exclamation mark did not generate a difference in the N1 component and alpha and theta ERSP did not correlate with the N1 component, suggesting that the differences of alpha and theta ERSP in the later time windows are not the consequences of differences in stimulus-driven activity. Detailed results of these analyses can be found in the Supplementary Information.

### Correlational analysis of task-related theta oscillatory activity

**Figure 4:**
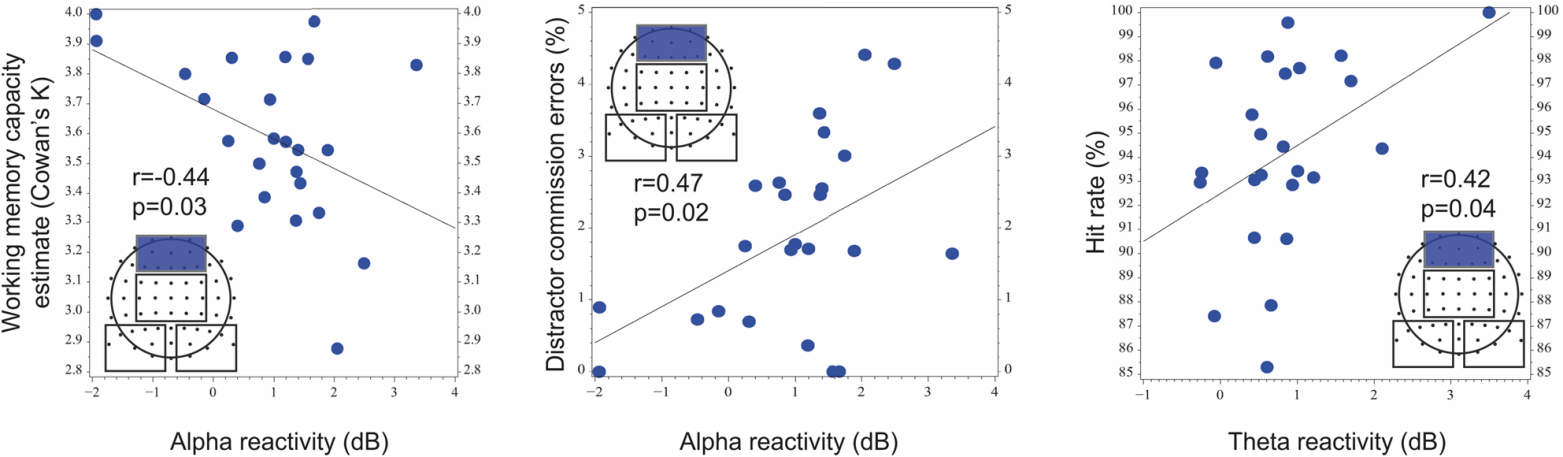
Correlation of frontal alpha and theta reactivity with behavioral results. We characterized alpha reactivity by the difference of alpha ERSP in the weak and strong distractor condition in the 700–1000 ms time window (alpha ERSP_weak distractor_—alpha ERSP_strong distractor_), while we characterized theta reactivity by the difference of theta ERSP in the learning and strong distractor condition in the 300–600 ms time window (theta ERSP_learning_—theta ERSP_strong distractor_). The Figure was prepared by using SAS 9.4 software.

To explore the relationship between the cognitive control-related modulation of frontal theta oscillatory activity and task performance, we characterized theta reactivity by the difference of ERSP in the learning and strong distractor condition. In these conditions the presented stimuli were similar except their color, indicating whether they should be learned or ignored. Theta reactivity in the frontal region showed a significant positive correlation with response accuracy (r = 0.42, *p* = 0.04) (Fig. 4). As one participant had extremely high theta reactivity, we performed the Spearman correlational analysis on the above-mentioned correlation as well, which is robust against the effect of outliers. The correlation between the theta ERSP and the hit rate became marginally significant (Spearman r = 0.39, *p* = 0.058). However, these results did not remain significant after correction for multiple comparisons.

## Discussion

### Alpha frequency band

During the retention period, regardless of the type of distractor, increased alpha ERSP was found following long sequences relative to short sequences. This memory load-related modulation of the alpha band is in line with many previous studies^4,38,66^. This effect remained significant in the central and left parieto-occipital region even after correction for multiple comparisons. The lateralization of memory load-related modulation is probably due to the phonological nature of the applied stimuli, which is generally processed in brain areas located in the left hemisphere^48–50^, which is also in line with former studies that used letters as memory items and detected more pronounced working memory load-related modulation in the left hemisphere^51,52^.

However, in contrast to the attributed inhibitory role of alpha enhancement during the increased need for distractor filtering^6,13^, alpha ERSP decreased with a higher level of distraction. Former studies examining alpha-band modulations during reactive filtering showed similar results^29,32^. Previous research on contingent capture showed, that salient distractors, that are similar to targets elicit an involuntary shift of spatial attention^67,68^. As the reduction of alpha power has been suggested as a marker of involuntary capture of attention^69^, attenuated alpha activity in the presence of a distractor that is highly similar to the targets might indicate the attraction of attention by the salient but distracting stimuli.

This interpretation is also supported by the positive correlation between alpha reactivity (i.e. stronger alpha ERD to strong relative to weak distractors) and the number of “distractor commission errors”, where participants mistakenly identified the previously presented distractor as a member of the memory sequence. This finding can be interpreted as the enhancing distraction of attention by the (stronger) distractor—indicated by a stronger ERD—increased the probability that it would be encoded as a member of the memory sequence, although we have to be aware that this correlation does not necessarily imply a causal relation. The alpha activity was mainly modulated by stimulus quality since stronger alpha ERD was observed in the presence of strong distractors relative to weak distractors (letters vs. exclamation mark), while no significant differences were found between the learning and strong distractor conditions (both stimuli were letters). This corresponds to a former study^32^, where reactive alpha modulation was present relative to target processing if the distractor had low similarity to the target stimulus, but not in the case of a highly similar distractor.

Therefore, in agreement with former studies^29,30,32^, our results suggest that in contrast to the inhibition of distractors outside of the attentional focus, alpha oscillatory activity is not directly involved in the rejection of task-irrelevant stimuli when they cannot be attentionally avoided.

Instead, decreasing and increasing alpha oscillatory activity might reflect the balance between externally and internally directed attention, respectively^70–72^. While alpha oscillatory power enhanced with the increasing internal processing demand of higher memory load, it attenuated (i.e. stronger ERD) with the increasing saliency of distractors and therefore the extent of involuntary capture of attention. Therefore, although alpha oscillations might serve as a top-down control mechanism to protect working memory maintenance^4^, the bottom-up interference of unexpected salient stimuli might attenuate this protection.

### Theta frequency band

Theta ERSP was significantly increased during the presentation of weak and strong distractors relative to the learning period. Frontal theta oscillation is an important marker of working memory processing^24^. Increased frontal theta oscillation has been consequently observed during working memory tasks related to increasing task demands^22–24^, but it has been also considered as a sign of successful memory manipulation, novelty, and conflict detection^8,73^. Therefore, recently it has been suggested, that frontal theta oscillatory activity is a potential general marker of cognitive control in tasks that involve uncertainty about actions and outcomes^7,27^. According to this framework, increased theta activity in response to unexpected distractors might signal a need for enhanced cognitive control during reactive filtering^7^.

Our results showed, that the appearance of weak and strong distractors were both accompanied by increased theta ERSP compared to the learning condition, which suggests that theta modulation is related to the general control processes during the task. Moreover, in line with former results^32^, salient distractors induced increased frontal midline theta activity compared to the non-salient distractor, which corroborates that theta modulation is related to the need for cognitive control^7^. However, this modulatory effect was not a robust phenomenon, as at the regional level it did not reach statistical significance.

The connection between theta modulation and cognitive control is also supported by the correlation between response accuracy and frontal theta reactivity related to reactive filtering, as the prefrontal cortex has been suggested as an important source of cognitive control in working memory tasks^74^.

### Limitations

There are some possible limitations of this study. First, our sampling method was non-random, as the participants were all university students, which limits the generalizability of our results. Moreover, the correlational analysis was done by following a ROI-based approach which can be a possible limiting factor as well.

Furthermore, although effect size and sample size are theoretically independent, the small sample size of our study might inflate effect sizes^75^, which is a further limitation. Therefore, further studies applying similar paradigms on larger and more representative samples are required to verify our preliminary results.

### Conclusion

Distractor rejection is accompanied by distinct underlying dynamics of oscillatory activity in the alpha and theta frequency band. Alpha ERSP is mainly modulated by the saliency of the presented stimuli. However, while alpha activity is not directly involved in the filtering mechanism, it reflects the vulnerability of attention to distracting salient stimuli.

In contrast to that, theta ERSP is modulated by the need for cognitive control when faced with unexpected distractors.

Furthermore, our results underline the need for the distinction of proactive and reactive filtering, as these processes are reflected by different oscillatory dynamics, regardless of their common intention^30^, although further studies applying similar paradigms are required to verify our results.

## Supplementary information

Supplementary Information.

## Data Availability

The datasets that are used and analyzed during the current study are available from the corresponding author on reasonable request.
