# Supplementary material for: Salient distractors open the door of perception: alpha desynchronization marks sensory gating in a working memory task
Source: Sci Rep. 2020 Nov 5;10:19179. doi: 10.1038/s41598-020-76190-3 (PMC7645677; doi:10.1038/s41598-020-76190-3)
Supplement: Supplementary file 1 — Supplementary Information. [file 41598_2020_76190_MOESM1_ESM.pdf]

Supplementary Information

Salient distractors open the door of perception: alpha desynchronization marks sensory gating in a working memory task

Zsuzsanna Fodor, MD[a], Csilla Marosi, MD[a], László Tombor, MD[a], Gábor Csukly, MD, PhD[a]

[a] Department of Psychiatry and Psychotherapy, Semmelweis University, Budapest, Hungary, 1083 Balassa utca 6.

Details of time-frequency analysis

The method described here generalizes the narrow-band measures of event-related synchronization and desynchronization [1] and includes both phase-locked and non-phase-locked contributions.

The principle of calculating the ERSP is to compute the power spectrum of the EEG signal from a sliding time window. For n trials, if $F_k(f,t)$ is the power of trial k at frequency f and time t, the ERSP value is calculated as

$$ERSP(f,t) = \frac{1}{n}\sum_{k=1}^{n} | F_k(f,t) |^2$$

To obtain the $F_k(f,t)$ function (the signal power at a given frequency and time point), the EEG signal was convolved with Hanning-windowed sinusoidal wavelets. The number of wavelet cycles increased evenly with frequency (starting at 0.6 cycles at 1.5 Hz) for optimal time-frequency resolution.

Effect of bottom-up differences in visual stimulation on the N1 component and correlational analysis with ERSP

In order to rule out that the detected distractor rejection-related modulatory effects of the ERSP were driven by differences in low level features we examined the N1 component and its correlation with alpha and theta ERSP.

According to former studies, early ERP components, such as the N1 are sensitive to the physical properties of the stimuli, index early sensory processing associated with higher level discrimination and the depth of early attentional capture and reflect the biological significance of the stimuli [2-5]. We assumed that if the distractor saliency-related modulation of the alpha activity is the consequence of the difference in stimulus-driven activity, this should manifest in the early ERP components as well.

Therefore, in order to investigate the stimulus-related bottom-up differences we compared the mean ERP of the Oz electrode in the strong and weak distractor conditions in the 90-155 ms time window (the conditions were averaged by memory load). The results showed that the type of the distractor (strong vs. weak) ($F(1,23) = 0.97$, $p = 0.34$) did not have an effect on the N1 component. Moreover, we performed the analysis in the Oz electrode with varying memory load in the strong and weak distractor conditions in the 90-155 ms time window. The results showed that neither the type of the distractor (strong vs. weak) (strong vs. weak) ($F(1,23) = 0.97$, $p = 0.34$) nor memory load (2-item vs. 6-item) ($F(1,23) = 1.95$, $p = 0.18$) had an effect on the N1 component.

Furthermore, we examined if there is a correlation between the N1 component and alpha and theta ERSP in the Oz electrode. The N1 component did not correlate significantly with alpha and theta ERSP in the weak distractor condition (alpha ($r = 0.14$, $p = 0.51$), theta ($r = 0.13$, $p = 0.56$)) and in the strong distractor condition (alpha ($r = 0.09$, $p = 0.68$), theta ($r = 0.11$ $p = 0.60$)).

We also performed the same correlational analysis on conditions with varying memory load. The N1 component did not correlate significantly with alpha ERSP in the weak distractor condition (low memory load ($r = 0.09$ $p = 0.68$) high memory load ($r = 0.18$ $p = 0.39$)) and in the strong distractor condition (low memory load ($r = 0.06$ $p = 0.78$) high memory load ($r = 0.13$ $p = 0.53$)). Furthermore, the N1 component did not correlate significantly with theta ERSP in the weak distractor condition (low memory load ($r = 0.06$ $p = 0.77$) high memory load ($r = 0.12$ $p = 0.59$)) in the strong distractor condition (low memory load ($r = -0.02$ $p = 0.94$) high memory load ($r = 0.06$ $p = 0.78$)) and in the learning condition (low memory load ($r = -0.04$ $p = 0.87$) high memory load ($r = 0.16$ $p = 0.46$)).

The N1 component was not affected by the exact stimulus properties and did not show significant correlation with alpha and theta ERSP in neither condition, suggesting that the differences of alpha and theta ERSP in the later time windows are not the consequence of differences in stimulus-driven activity.

Supplementary Figure 1

Theta ERSP in learning, strong and weak distractor conditions.

Theta event-related spectral perturbation (ERSP) in strong and weak distractor conditions (300–600 ms time window highlighted). The Figure was created using MATLAB and Statistics Toolbox Release 2017a.

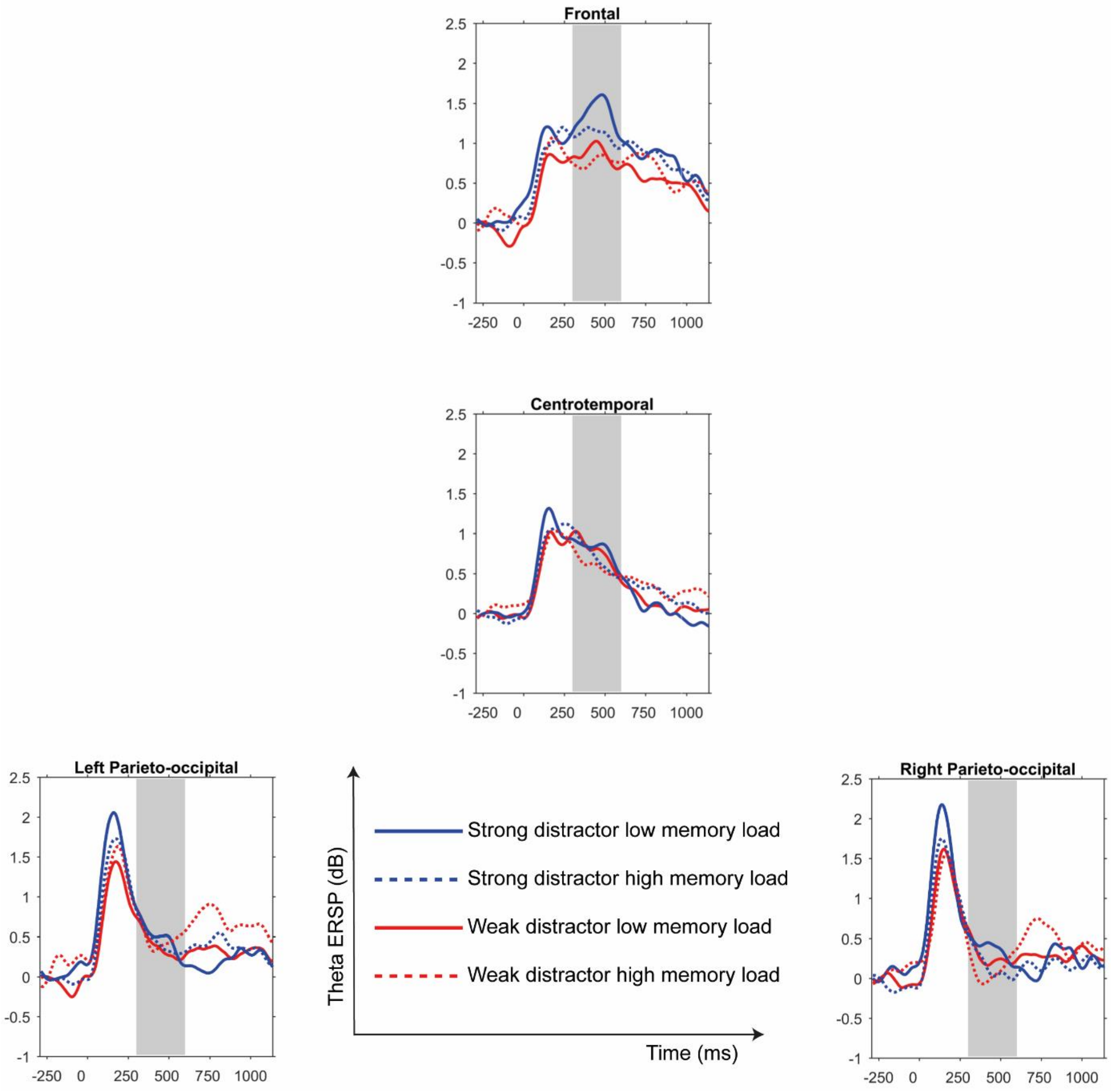

Supplementary Figure 2.
Alpha ERSP in learning, strong and weak distractor conditions.
Alpha event-related spectral perturbation (ERSP) in strong and weak distractor conditions with error envelopes (700–1000 ms time window highlighted). The Figure was created using MATLAB and Statistics Toolbox Release 2017a.

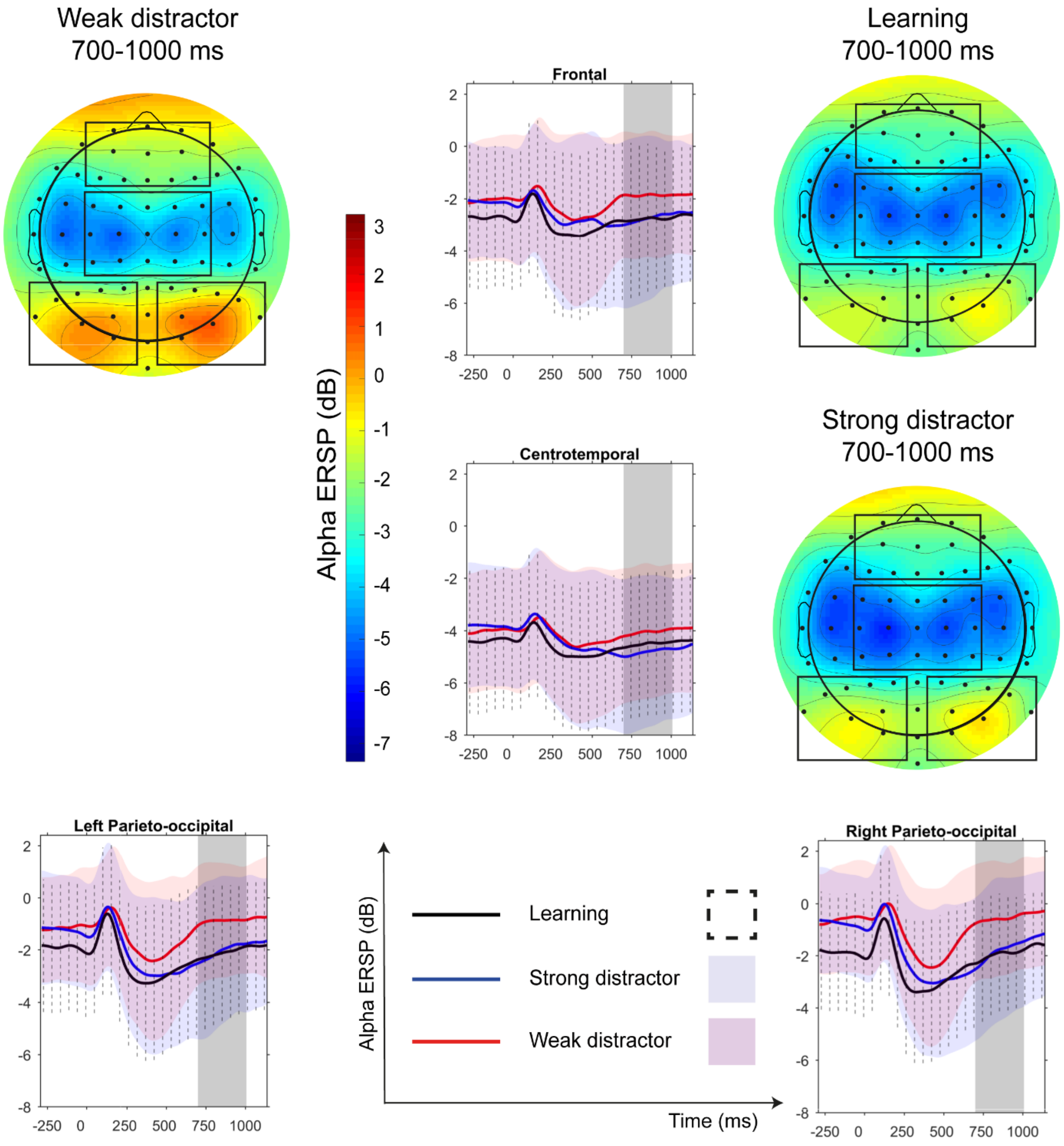

Supplementary Figure 3.
Alpha ERSP in strong and weak distractor conditions with error envelopes.
Alpha event-related spectral perturbation (ERSP) in strong and weak distractor conditions with error envelopes (700–1000 ms time window highlighted). The Figure was created using MATLAB and Statistics Toolbox Release 2017a.

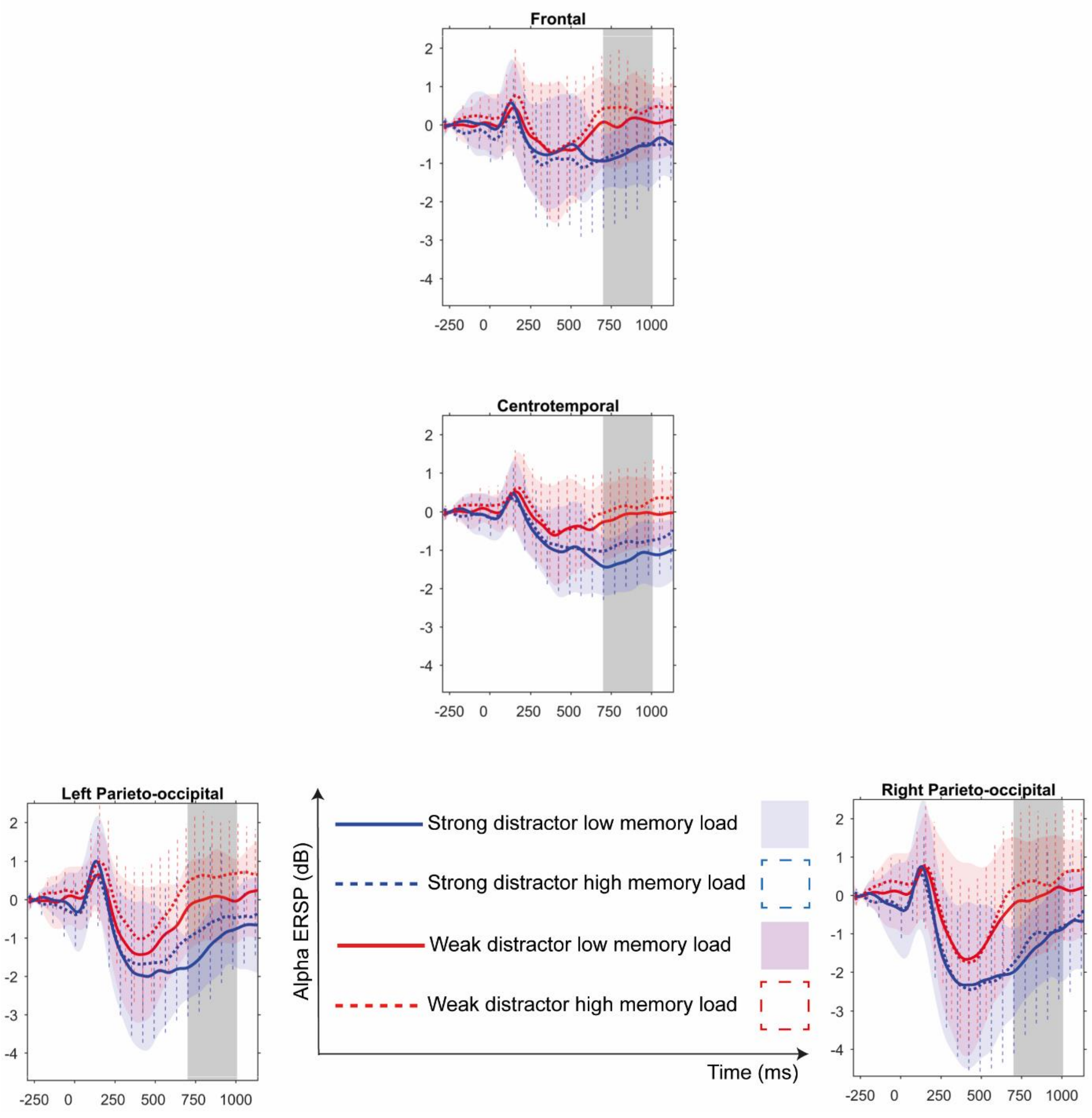

Supplementary Figure 4.

Theta ERSP in learning, strong and weak distractor conditions with error envelopes.

Theta event-related spectral perturbation (ERSP) in learning, strong and weak distractor conditions with error envelopes (300–600 ms time window highlighted). The Figure was created using MATLAB and Statistics Toolbox Release 2017a.

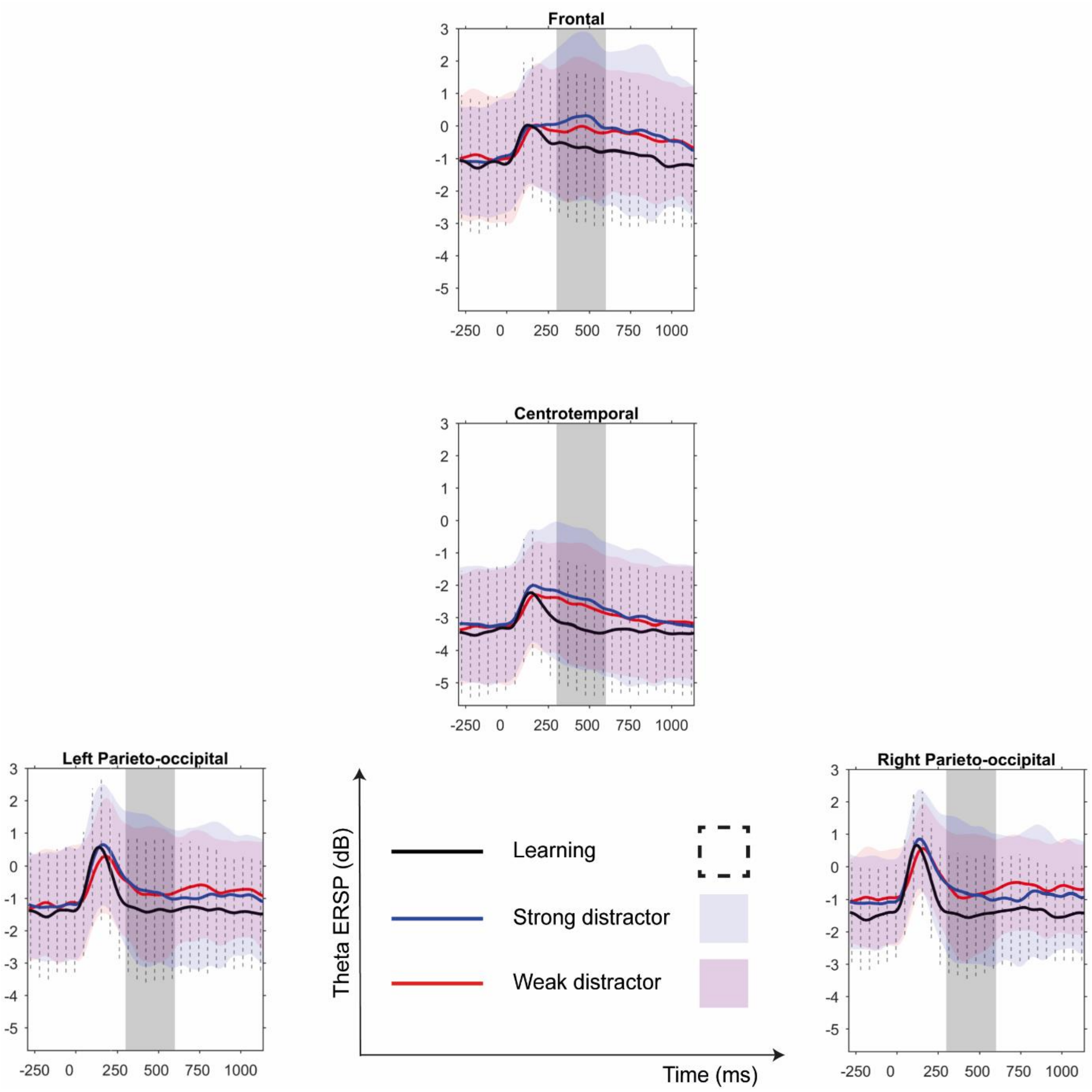

REFERENCES

1 Pfurtscheller, G. & Aranibar, A. Event-related cortical desynchronization detected by power measurements of scalp EEG. *Electroencephalogr Clin Neurophysiol* **42**, 817-826 (1977).

2 Ligeza, T. S., Tymorek, A. D. & Wyczesany, M. Top-down and bottom-up competition in visual stimuli processing. *Acta neurobiologiae experimentalis* **77**, 305-316 (2017).

3 Vogel, E. K. & Luck, S. J. The visual N1 component as an index of a discrimination process. *Psychophysiology* **37**, 190-203 (2000).

4 Hart, S. J., Lucena, N., Cleary, K. M., Belger, A. & Donkers, F. C. Modulation of early and late event-related potentials by emotion. *Frontiers in integrative neuroscience* **6**, 102, doi:10.3389/fnint.2012.00102 (2012).

5 Luck, S. J. *An Introduction to the Event-Related Potential Technique*. (MA: MIT, 2014).
